# Beyond the map: evidencing the spatial dimension of health inequalities

**DOI:** 10.1186/s12942-020-00242-0

**Published:** 2020-11-09

**Authors:** Fayet Yohan, Praud Delphine, Fervers Béatrice, Ray-Coquard Isabelle, Blay Jean-Yves, Ducimetiere Françoise, Fagherazzi Guy, Faure Elodie

**Affiliations:** 1 Equipe EMS – Département de Sciences Humaines et Sociales, Centre Léon Bérard, 28 rue Laennec, 69008 Lyon, France; 2 EA 7425 Health Services and Performance Research, Université de Lyon, Lyon, France; 3 Department Prevention Cancer Environment, Centre Léon Bérard, Lyon, France; 4 Inserm UA 08: Radiations, Défense, Santé, Environnement, Centre Léon Bérard, Lyon, France; 5 Department of Medical Oncology, Centre Léon Bérard, Université Claude Bernard, Lyon, France; 6 Digital Epidemiology and e-Health Research Hub, Department of Population Health, Luxembourg Institute of Health, Strassen, Luxembourg; 7 Center of Epidemiology and Population Health, UMR 1018, Inserm, Paris South, Paris Saclay University, Villejuif, France; 8 Gustave Roussy Institute, Villejuif, France

**Keywords:** Health inequalities, Environment, Social deprivation, Health care access, Geography, Public health, France, GIS

## Abstract

**Background:**

Spatial inequalities in health result from different exposures to health risk factors according to the features of geographical contexts, in terms of physical environment, social deprivation, and health care accessibility. Using a common geographical referential, which combines indices measuring these contextual features, could improve the comparability of studies and the understanding of the spatial dimension of health inequalities.

**Methods:**

We developed the Geographical Classification for Health studies (GeoClasH) to distinguish French municipalities according to their ability to influence health outcomes. Ten contextual scores measuring physical and social environment as well as spatial accessibility of health care have been computed and combined to classify French municipalities through a K-means clustering. Age-standardized mortality rates according to the clusters of this classification have been calculated to assess its effectiveness.

**Results:**

Significant lower mortality rates compared to the mainland France population were found in the Wealthy Metropolitan Areas (SMR = 0.868, 95% CI 0.863–0.873) and in the Residential Outskirts (SMR = 0.971, 95% CI 0.964–0.978), while significant excess mortality were found for Precarious Population Districts (SMR = 1.037, 95% CI 1.035–1.039), Agricultural and Industrial Plains (SMR = 1.066, 95% CI 1.063–1.070) and Rural Margins (SMR = 1.042, 95% CI 1.037–1.047).

**Conclusions:**

Our results evidence the comprehensive contribution of the geographical context in the constitution of health inequalities. To our knowledge, GeoClasH is the first nationwide classification that combines social, environmental and health care access scores at the municipality scale. It can therefore be used as a proxy to assess the geographical context of the individuals in public health studies.

## Background

Health inequalities have emerged as a political challenge in many developed countries [1] but few interventions have been sufficient to address this issue, the scope of which extends well beyond public health [2]. While social inequalities have been explored comparing health outcomes according to the social characteristics of the individuals [3], maps [4] and contextual studies [5] have raised the question of the spatial dimension of health inequalities. For example, standardized mortality rates in France were two times higher in Northern counties compared to the South-East in the early 2000’s [6]. At a finer spatial scale, maps have also reported better health outcomes in urban areas than in the surrounding rural areas as well as other inequalities according to neighborhoods within urban areas [7]. The breadth of spatial inequalities in health and the continuity of trends across time show that these inequalities do not occur by chance. We assume they reflect the unequal distribution of health risk according to the characteristics of the geographical contexts.

### Combined effects of local health care accessibility, environmental and social characteristics lead to spatial inequalities in health

Three types of contextual risk factors involved in a combined manner in the constitution of spatial inequalities in health can be outlined from the literature:

- Physical environments: Studies considering physical environment generally focused on exposure to a single environmental exposure such as air pollution [8], noise [9], water contaminants [10], ultraviolet radiation [11] or green spaces [12]. Moreover, environment contributes also to healthy behaviours, raising the question of urban planning [13] and food environment [14].
- Social characteristics of the population: This dimension is well-known thanks to deprivation indices developed at a small area level by social epidemiology, such as in the United Kingdom [15, 16] or in France [17, 18]. Multilevel analyses in health research in the 1990s found deprivation to impact on health outcomes, whilst taking into account individual characteristics [19]. This demonstrates that social context must be also considered in the analysis of spatial inequalities in health.
- Spatial accessibility to health care: Studies demonstrated a lower health care use in lower medical density areas [20] and for remote patients [21]. Health care access may improve prevention and early diagnosis of diseases but also follow-up and management [22]. Assessment of health care accessibility must also take into account the quality of care according to the facilities, their size and level of expertise. In the case of cancer care for example, remote patients having a lower accessibility to expert centers, suffer from poorer health care and consequently poorer survival rates [23].

These determinants of health suggest the geographic contexts’ potential to influence health status and to produce health inequalities. However, estimating the impact of the geographic context on health inequalities remains difficult, partly because epidemiological studies aiming to identify associations between spatial characteristics and health outcomes most often investigate one spatial factor at a time, according to the objectives of the study. As a result, geographic contexts may be variously measured, in terms of characteristics and spatial scale. In addition, spatial indices may be combined differently across studies, due to methodological choices and study objectives. Consequently, studies analyzing geographical inequalities in health are based on different geographical frameworks limiting the comparability of the results [24]. Furthermore, the separated analysis of risk factors in epidemiological studies mostly impedes a comprehensive review of all the vulnerabilities related to the place of residence. Some epidemiological studies using a social deprivation index took this limit into account, investigating a potential difference in their analysis between rural and urban deprived areas [25]. Considering this challenge, developing a geographical classification is needed to assess these combined effects of geographical determinants on health and to develop a common geographical frame of reference in health studies. This paper aims to demonstrate the value of this new comprehensive classification approach to address the geographical context’s contribution into health inequalities. The “Geographical Classification for Health studies” (GeoClasH) was computed from spatial data measuring physical environment, social deprivation and health care accessibility, using the example of mainland France.

## Methods

We used spatial data available for the 35,798 mainland France municipalities regarding environmental factors, social environment and spatial accessibility of the health care. Ten geographical scores have been computed from these data and combined to classify French municipalities through K-means clustering. As a gold standard to study health inequalities, age-standardized all-causes Mortality Rates (SMR) have been calculated to compare the general health status according to the clusters of the GeoClasH classification.

### Geographical data and scores calculation

The year of reference of the geographical data used in the classification was 2014 corresponding to the date of the last detailed French census data, provided by the National Institute for Statistics and Economic Studies (INSEE). Our study deals only with mainland France because little data were available for French overseas territories. We used the GeoFLa municipalities basemap from the National Geographic Institute (IGN) for 2016, which comprises 35,798 municipalities. For all spatial data, the projection used was the Lambert 93 being the current projection system in effect in France. As this study aims to evidence the spatial dimension of health inequalities at a fine scale, only variables measuring the contextual features and exhaustively available at the municipality scale in mainland France were selected (Table 1).

**Table 1:** Contextual features involved in spatial inequalities in health and variables included in the design of the GeoClasH classification

Contextual features	Variables	Time	Data sources
Physical environment	Population density	2014	IGN-INSEE
	Air quality score	2009–2013	INERIS
	Pesticides’ risk exposure	2012	Corine Land Cover
Social characteristics of the population	Median income per household people	2014	INSEE
	Unemployment rate	2014	INSEE
	Percentage of labourers and employees	2014	INSEE
	Percentage of high school graduates	2014	INSEE
	Percentage of unattached individuals	2014	INSEE
Spatial accessibility to health care	Average journey time to hospital	2014	SAE-Odomatrix
	Spatial accessibility to primary care practitioners	2013	DREES

Every variables included in the calculation of the GeoClasH classification was produced at the municipality scale

Variables considered but unavailable at the municipality scale: water quality; noise; ultraviolet radiation; green spaces; food, alcohol and tobacco retail environments

For confidentiality reasons, because of their low population some data were missing in some low-populated municipalities. Low population may also trigger extreme values (in the rates calculation for example) which may lead to a bias in the multivariate analysis used to design the classification process if there were used in the multivariate analysis. To overcome this issue, variables with missing values at the municipality scale were also calculated at the “canton” scale. A canton in France is a continuous electoral district essentially defined on demographic bases and its scale is slightly larger than the Commune scale. There were 1989 cantons across the mainland France and 35,789 municipalities in 2014. The canton value was assigned to municipalities with missing data or with a reference population of less than 50 inhabitants.

#### Environmental scores

##### Population density

The population density was calculated for 2014 using the surface area in km^2^ of municipalities from the IGN’s GEOFLA database, and the 2014 number of inhabitants per municipalities from the population censuses provided by INSEE. Thus, for each municipality, a population density per km^2^ has been computed.

##### Air quality

Data provided by the French National Institute for Industrial Environment and Risks (INERIS) on particulate matters (PM_10_ and PM_2.5_) and nitrogen dioxide (NO_2_) were used to estimate the average exposure to each pollutant for each municipality between 2009 and 2013, in accordance with methods reported by Pascal et al. [26]. To elaborate the synthetic air quality score over the period 2009–2013, we considered the different spatial distribution of particulate matters in comparison of NO_2_ which are more related to road traffic (Additional file 2: Fig. S2). We first computed for each municipality the mean value of PM_2.5_ and PM_10_ concentration in µg/m^3^ as *PM *= *mean(PM*_*2.5*_*;PM*_*10*_*)*. Then, the air quality score was calculated, for each municipality, as the mean of PM and NO_2_ as follows: *Air quality score *= *mean(NO*_*2*_*;PM)*.

##### Risk of pesticides’ exposure

Due to the lack of data on pesticides applications and types used in France, we used the 2012 Corine Land Cover nomenclature to assess pesticide exposure level at the municipality scale, using the share of the municipalities’ area occupied by crops using pesticides (on-irrigated arable lands, vineyards and orchards) as a proxy for exposure [27].

#### Social environment scores

We considered socio-economic variables used in the most sensitive deprivation index developed for France [28] and published by Rey et al. [18]:

- the median income per household people
- the unemployment rate in the population aged 15 to 64 years
- the percentage of labourers and employees in the population aged 15 to 64 years
- the percentage of high school graduates in the population aged 15 years and older
- the percentage of unattached individuals in the population aged 15 years and older.

#### Health care access scores

##### Spatial accessibility to primary care practitioners

We used the reference indices to assess the spatial accessibility of healthcare practitioners in France [29, 30]: the APL (*Accessibilité Potentielle Localisée*, localized potential accessibility) indicators measuring spatial accessibility to primary care practitioners (general practitioners, nurses, chemists, gynecologists, midwives, physiotherapists, dentists, ophthalmologists, pediatricians and psychologists). We computed a synthetic score of the primary care accessibility at the municipality scale. Considering the importance of general practitioners in the primary care system, their APL indices count for half in the calculation of the final score, measuring the accessibility to primary care. We used principal component analysis (PCA) to summarize the other primary care practitioners’ indices and selected the first component (accounting for 45% of the variance in the dataset) of the PCA as the score of these practitioners’ accessibility. The final score was the mean value of both accessibility scores of general practitioners and other primary care practitioners.

##### Spatial accessibility to hospitals

French municipalities hosting a hospital (n = 536 municipalities) were identified using the Annual Statistics of the Facilities (SAE) database (year 2014), which is managed by the French ministry of health. To measure the municipalities’ remoteness to hospitals, journey times by car to the closest municipality hosting a hospital were estimated using the Odomatrix^®^ software (Source: ODOMATRIX, INRA UMR1041 CESAER, from IGN Route500^®^).

### Preliminary analysis of the dataset and clustering process of the French municipalities

A correlation matrix was performed using the R software (version 3.5, package “Corrplot”) to study potential correlation between the selected variables and to justify the relevance of each variable to be included in the clustering process. Several methodologies are used to develop multivariate classification of areas [31]. Hierarchical clustering and partitioning methods have their limitations but hierarchical clustering is known to be more computationally demanding and less efficient than partitioning methods with large datasets [32]. Considering our large dataset (ten variables, 35,798 municipalities), we selected the K-means partitioning algorithm using the R software (version 3.5, packages “NbClust”) to perform the classification. Geographical variables were standardized using the R’s “scale” function, before incorporating them into the clustering process. We performed several K-means partitioning from the same dataset in order to ensure the clustering process’ stability and the obtained classification was always the same. While the classification aims to allow health outcomes’ comparison across geographical contexts, we were careful not to deal with too heterogeneous clusters considering their population size in order to be statistically comparable. We chose therefore the five-cluster partitioning because the six-cluster partitioning splits the RM (Rural Margins) cluster in two parts whereas the population size of this cluster was already the lowest.

### Calculation of the age-standardized mortality rates according to the GeoClasH clusters

In order to assess the relevance of the classification to display health inequalities, the age-standardized mortality ratio (SMR) and their 95% confidence interval (CIs) was calculated in each cluster, as the ratio of observed deaths and the expected deaths in each cluster based on the distribution of mainland France, using the indirect method. The expected deaths were based on age-specific deaths in the mainland France. We obtained data on overall population and mortality from the INSEE databases, for the mainland France at the municipality scale, in 5-year age groups (i.e. 0–4, 5–9… 80–84, and ≥ 85 years) from 2011 to 2015 [33]. Data were not available at the municipality scale to standardize also on sex distribution.

We interpreted the SMR using their CI; we considered that observed mortality rates were significantly different to expected mortality rates (based on mainland France rates) when SMRs were significantly greater than 1. We considered SMRs as significantly different between each other when their CIs did not overlap.

## Results

**Fig. 1:**
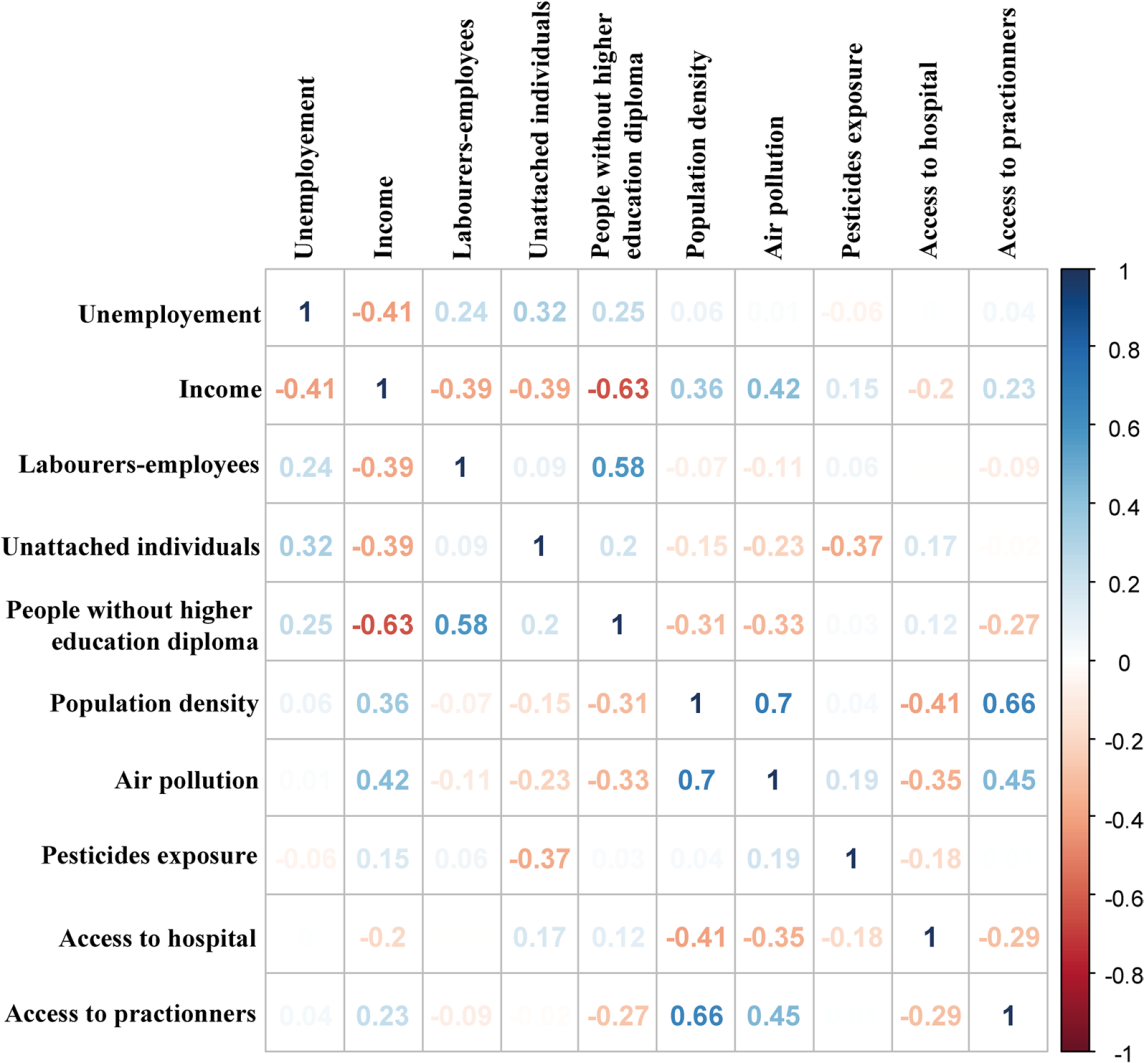
Correlation matrix of the selected variables, Mainland France

The maps of the ten geographical scores included in the GeoClasH classification are provided in Additional files to describe and improve the understanding of these scores, giving an overview of spatial inequalities in France (see Additional files 1, 2, 3, 4, 5, 6, 7, 8, 9 and 10). The correlation matrix (Fig. 1) reports some moderated correlations for population density with air pollution (cor = 0.7) and access to practitioners (cor = 0.66), as well as between income and people without higher education diploma (cor = 0.63). But we consider these correlations are not strong enough to exclude one of these variables, which each have their own relevance and interest with regards to literature.

**Fig. 2:**
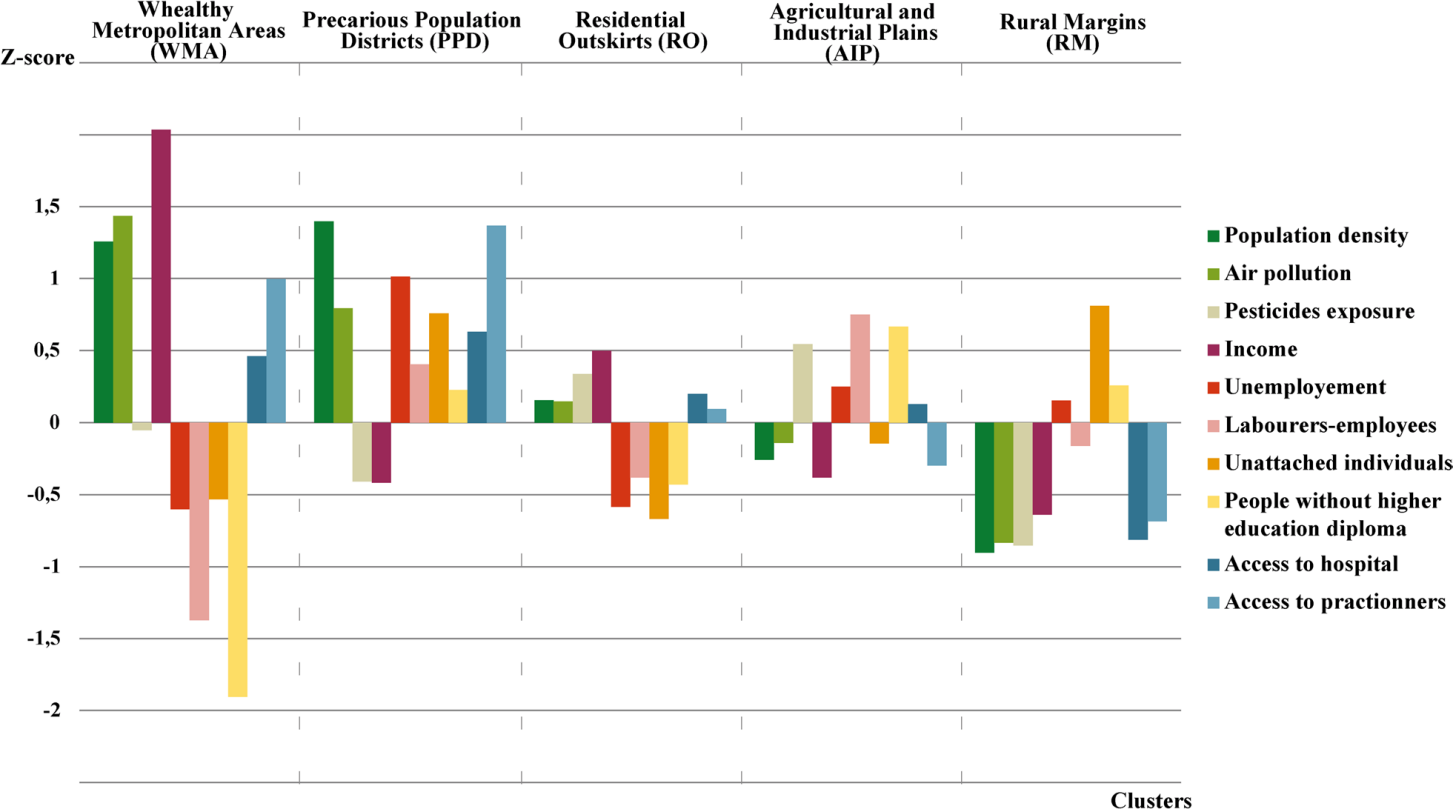
Mean z-score for each of the variables within the clusters, GeoClasH, Mainland France

**Fig. 3:**
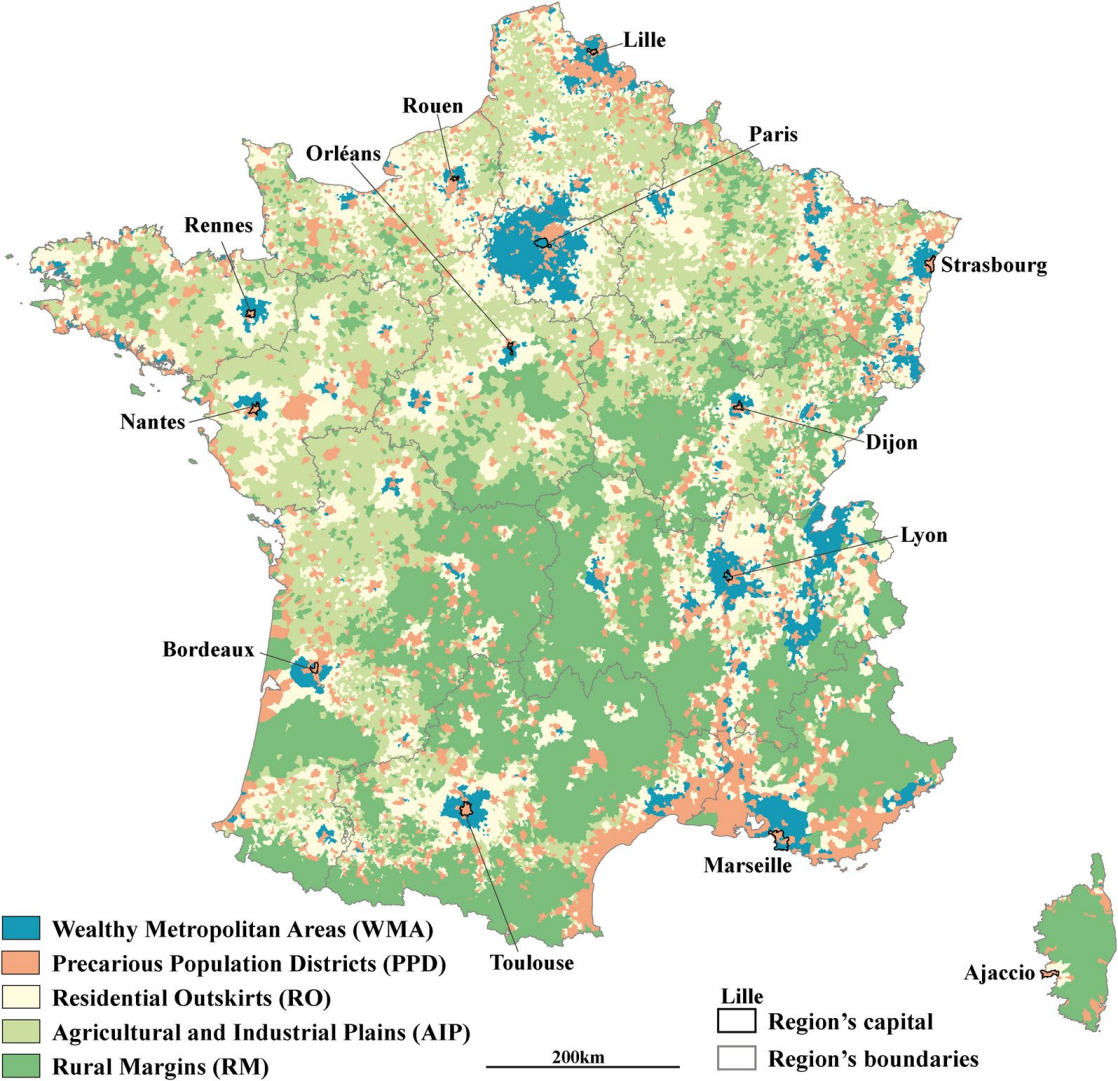
Geographical distribution of the clusters in the Mainland France, GeoClasH

Average values (Table 2) and z-score means (Fig. 2) for each of the geographical variables used in the classification process have been calculated and compared to characterize the clusters. The geographical distribution of the clusters (Fig. 3) was considered and used to name them.

**Table 2:** Main characteristics of the five clusters, GeoClasH, Mainland France

Characteristics, mean (SD)	Wealthy Metropolitan Areas (WMA)	Precarious Population Districts (PPD)	Residential Outskirts (RO)	Agricultural and Industrial Plains (AIP)	Rural Margins (RM)	France
Population density (pop/km^2^)	813 (2793)	701 (1496)	77 (74)	43 (36)	19 (21)	178 (973)
Exposure to NO_2_ (µg/m^3^)	17.0 (6.0)	14.2 (5.4)	10.9 (2.8)	9.5 (2.3)	6.5 (2.2)	10.3 (4.4)
Exposure to PM (µg/m^3^)	19.4 (1.9)	18.6 (1.7)	18.2 (1.2)	18.0 (1.2)	17.1 (0.9)	18.0 (1.4)
Share of the area occupied by crops using pesticides (%)	34.0 (28.4)	23.3 (22.9)	45.7 (29.5)	52.0 (26.0)	10.0 (15.5)	35.6 (30.0)
Unemployment rate (%)	6.2 (1.7)	11.2 (2.9)	6.3 (1.9)	8.8 (2.6)	8.5 (3.3)	8.0 (3.0)
Annual income per household (€)	26,050 (3372)	19,162 (1750)	21,733 (1794)	19,270 (1376)	18,543 (1722)	20,335 (2808)
Labourers-employees (%)	38.4 (8.6)	59.1 (7.5)	50.0 (9.0)	63.2 (8.5)	52.5 (10.8)	54.4 (11.7)
Unattached individuals (%)	11.7 (4.3)	18.2 (5.1)	11.1 (3.0)	13.7 (3.5)	18.5 (4.7)	14.4 (5.3)
People without higher education diploma (%)	63.0 (8.1)	79.9 (5.8)	74.7 (5.3)	83.4 (4.3)	80.2 (6.2)	78.1 (7.9)
Journey time to hospital (min)	18.5 (8.5)	16.5 (10.4)	21.6 (8.3)	22.4 (8.2)	33.5 (14.2)	23.9 (11.7)
APL score to general practitioners	57.7 (22.9)	84.4 (33.4)	45.7 (26.2)	41.7 (24.7)	31.5 (31.9)	46.1 (31.6)
Number of municipalities	2920	3742	10159	10330	8647	35798
Overall population	14,435,670	32,023,919	8,717,397	5,902,766	2,948,094	64,027,846

SD: Standard deviation, NO_2_: Nitrogen dioxide, PM: particulate matter, APL: localized potential accessibility

Wealthy Metropolitan Areas (WMA) stand out from the other clusters for their higher population density, very low social deprivation and good spatial accessibility to health care. This cluster has the most pronounced profile (Fig. 2) and includes municipalities hosting the wealthiest populations of major French urban areas, who benefited from their economic dynamism but are also exposed to their drawbacks (air pollution). Depending on local configurations, these municipalities may not be located in the center of urban areas but rather in the near periphery. Precarious Population Districts (PPD) are densely populated municipalities with the best spatial accessibility to health care. Contrary to the WMA, most of the social deprivation indices report the social vulnerabilities of the PPD population. Lower access to practitioners in the WMA in comparison to PPD can be linked to the higher cost of life and housing in these areas that can dissuade the establishments of practitioners. The PPD cluster is the biggest cluster in terms of population (more than 32 million inhabitants) and encompasses some downtowns or working-class suburbs of the biggest cities, previous and current industrial districts and most of the little and medium-sized cities.

Residential Outskirts (RO) are situated around the urban areas and have a rather intermediate profile with regard to spatial accessibility to health care and to the environment, except for exposure to pesticide risk, probably due to the proximity of countryside and agricultural activities. This population is rather privileged compared to the national average. Agricultural and Industrial Plains (AIP) are rural spaces strongly marked by productive activities in terms of population (63% of laborer or employee, 83% of people without higher education diploma) and environmental exposures (risk of pesticides exposure). This cluster includes most of the countryside municipalities in the North of France and some valleys in the South. Rural Margins (RM) are remote spaces far from urbans dynamics and stresses. The population of this cluster benefits from the quality of its environment (fewest exposures to air pollution and pesticides) but experiences social deprivation (lowest average income and highest rate of unattached individuals) and difficulties to get access to health care services (worst indices regarding the health care access). Most of these municipalities are situated in relief or mountainous areas.

Table 3 gives observed and expected numbers of deaths and the corresponding SMRs with their 95% confidence intervals for each cluster. The number of deaths was significantly lower than expected in the WMA (95,440 vs 109,929, SMR = 0.868, 95% CI 0.863–0.873) and in the RO (63,206 vs 65,106, SMR = 0.971, 95% CI 0.964–0.978) compared to the mainland France population. Significant excess mortality were found for PPD (SMR = 1.037, 95% CI 1.035–1.039), RM (SMR = 1.042, 95% CI 1.037–1.047) and AIP (SMR = 1.066, 95% CI 1.063–1.070) compared to the mainland France population.

**Table 3:** Age-standardized mortality ratios (SMR) according to the GeoClasH clusters, France, 2011–2015

	Population	Observed deaths (rate)	Expected deaths (rate)	SMR (95% CI)
Wealthy Metropolitan Areas (WMA)	14,436,150	95,440 (0.66%)	109,929 (0.76%)	0.868 (0.863–0.873)
Residential Outskirts (RO)	8,716,474	63,206 (0.73%)	65,106 (0.75%)	0.971 (0.964–0.978)
Precarious Population Districts (PPD)	32,024,097	300,523 (0.94%)	289,795 (0.90%)	1.037 (1.033–1.041)
Rural Margins (RM)	2,947,557	35,969 (1.22%)	34,525 (1.17%)	1.042 (1.031–1.053)
Agricultural and Industrial Plains (AIP)	5,903,512	59,044 (1.00%)	55,365 (0.94%)	1.066 (1.058–1.075)

## Discussion

This paper shows the potential of geographical context to influence health outcomes and its significant involvement in the constitution of health inequalities. As the Marmot Review recommended to fully integrate planning, transport, housing, environmental and health systems to address the social determinants of health in each locality [1], the GeoClasH classification supports a comprehensive understanding of the spatial dimension of health inequalities. Based on ten scores measuring local health care accessibility, environmental and social characteristics, the GeoClasH classification is, to our knowledge, the first nationwide geographical classification designed for health studies at the municipality scale combining these different contextual features impacting health outcomes. Indeed, geographical indicators combining several features are usually calculated as a continuous variable [34] and often include health outcomes variables in their calculation. As an example, the Index of Multiple Deprivation (IMD) includes an “Health Deprivation and Disability Domain”, based on morbidity, disability and premature mortality measures, into its score calculation [24, 35]. Combining health outcomes and spatial data in the calculation of the index can aid policy-makers identify the most deprived areas in need of priority public health interventions [36, 37] while the experimental GeoClash classification has the specific objective to assess the contribution of geographical context in the constitution of health inequalities. The classification allows the comparison of populations that are heterogeneously exposed to health risks because of their geographical context.

Using the clustering process is relevant to distinguish and address areas of distinct characters, as specific combination of geographical features, since previous studies have already shown it [38–40]. This multidimensional and combined approach is expected to have a strong beneficial impact on future epidemiological studies to estimate the environmental context of the individuals in the larger sense of the term. Completing social deprivation indices, the GeoClasH classification could therefore be used to provide a simple and comprehensive assessment of the geographical context of the studied subjects and to document health inequalities. It can also be useful for multi-level analyses, for adjusting regression models or leading subgroup analyses. The clustering process used avoids the current limitations of spatial analysis at a fine geographic scale, like the modifiable area unit problem [41] since municipalities are aggregated according to their own characteristics and not according to their surface area or proximity. The classification can be used for recent years but can also be valid for last decades due to small changes and constant trends in the French geographical area regarding variables included in the classification. Our methodology can be potentially reproducible in other countries and scalable with different number of clusters, in accordance with the dataset. As an example, air pollution data used in this study can be obtained in other European countries as well as the data used to estimate the probability of pesticide use [42].

Our results report significant health inequalities according to the GeoClasH clusters and especially the positive situation of Wealthy Metropolitan Areas (WMA) in comparison to mainland France, with a 15.1% lower mortality. These results are particularly remarkable because the GeoClasH classification was only designed on theoretical basis to demonstrate the complex constitution of spatial inequalities in health which result from the combining effects of local health care accessibility, environmental and social characteristics. Indeed, the ability of the GeoClasH classification to evidence the real extents of spatial inequalities in mortality in France could have been much better if we could have overtaken some limitations. First, the GeoClasH classification considers each geographical variable equally while each one probably does not have the same impact on health outcomes. Weighting the contribution of each variable in the clustering process according to their real influence on mortality can help to improve the quality of the classification for example. But this information is currently not available in the literature. Studies that would split the contribution of each major geographical variable may be helpful to improve our understanding of the contextual potential to influence health and the precision of geographical classification used in health studies. Considering the geographical scale, we may have chosen the IRIS (an acronym of ‘aggregated units for statistical information’) scale, the infra-municipal scale produced by the INSEE. But only social data are available at this scale. An IRIS scale classification would perhaps have been more sensitive to social disparities inside urban areas, and deprivation indices in France are already developed to precisely study social inequalities inside the urban areas [17, 18]. Finally, protection of individuals’ privacy supported restrains the use of IRIS scale in health studies because collection of identifying data as patients’ address is mostly limited by the independent data protection authority in France (“Commission nationale de l’informatique et des libertés”, https://www.cnil.fr/en/home). We have therefore opted for the municipal level to protect the balance of the GeoClasH classification and promote its compatibility and its implementation with most health studies.

Among the variables considered, only data with good temporal consistency and with a sufficiently precise scale has been used. Therefore, water quality data have not been integrated because of the low number of sampling in sparsely populated areas. For example, weekly biological measurements were made in some densely populated areas while just one value in 5 years was available for sparsely populated areas. The low number of samplings raises question about the representativeness of these few measurements and could also introduce a potential bias between urban and countryside municipalities. We only had annual data on PM and NO_2_ concentrations and it would be interesting to use hourly data to consider the number of threshold exceedances that are known to have short term effects on health [43]. Corine Land Cover data used to estimate environmental exposures to pesticide were not initially designed to this purpose even if several studies recognized Corine Land Cover data as the most suitable database available for national scale studies [27, 44]. Crop density by municipality has been used in ecological studies as a surrogate for residential exposure to agricultural pesticides [45]. Each plot (25 ha) has been characterized by its most extensive crop that may lead to not considering small specific acreage within the plot. Data to provide a nationwide measure of food, alcohol and tobacco retail environments are not currently available in France [46]. Similarly, we do not currently have consistent, reliable and comprehensive data in France to measure spatial coverage dedicated to greenspaces [12, 13]. As developing countries can lack from freely available data, the use of data from satellite images can be considered as interesting option to measure the physical environments. For example, nighttime lights can be considered as a relevant surrogate of the level of urbanization.

Social deprivation scores are generated from national census data but it does not take into account some local initiatives led by associations, foundations, local policies or management networks dedicated to specific diseases that have been implemented in some deprived areas (especially in urban areas) to improve behaviors and health care accessibility in its multiple dimensions (spatial, cultural, financial, etc.). Other dimensions of the health care spatial accessibility may have been taken into account including the different level of expertise of technical equipment according to the facilities [47]. While telemedicine has been recently implemented, its use and potential to improve spatial accessibility of health care access could be taken into account and regularly updating the GeoClasH model over time will help to evaluate the impact of such major changes in healthcare organization.

Despite these limitations and although the classification was designed a priori, significant mortality inequalities between the clusters have been emphasized. These results highlight the potential of the geographical context to promote health outcomes and its relevance in the study of health inequalities. It also evidences the extent of health inequalities according to geographical contexts, which due to their characteristics, are more or less favorable to health. Despite some environmental exposures whose impact on health outcomes is clearly demonstrated, WMA appear to be the most favorable context to health. This finding underscores the importance of making a strong distinction between studies on health inequalities and determinants to avoid confusion and design specific policies [48]. Studies in health determinants are seeking to evaluate the specific impact of each determinant on health at the individual scale. Identifying the causes of significant differences in health outcomes at the population level is required to analyze the constitution of health inequalities. As epidemiological research and general interest are increasing on environmental exposures and their impacts on health, our results confirm that spatial inequalities in health express a more complex process through the combined effects of many contextual features.

Variable availability of spatial data in different countries can currently limit the identical reproduction of this nationwide classification of the municipalities, even if more data can be available in urban areas. Moreover, this study considering the combined effects of local health care accessibility, environmental and social characteristics promotes a comprehensive approach which can be implemented everywhere to better understand the constitution of these inequalities and to support the design of relevant actions to efficiently reduce it. Nationwide exhaustive data as well as compatible and cross-sectional analyses are needed to improve knowledge of geographical inequalities in health and the understanding of its underlying mechanisms, in order to support evidence-based public policies addressing these major public health issues. Using the same geographical referential in health studies will make possible to compare gaps between health outcomes, and to define which outcomes are more determined by the geographical context. Moreover, it can contribute to identify and target specific public health issues to each geographical context. The GeoClasH classification can be used as a general contextual proxy that will be useful and relevant to assess the geographical context of individuals in public health studies and it support also dedicated research to efficiently address the spatial inequalities in public health.

## Conclusions

The Geographical Classification for Health studies (GeoClasH) was developed to demonstrate the value of a new comprehensive classification approach to evidence the spatial dimension of health inequalities. To our knowledge, this is the first nationwide classification that combines social, environmental and health care access scores at the municipality scale. Our results report significant inequalities in age-standardized mortality according to the GeoClasH clusters and especially the positive situation of Wealthy Metropolitan Areas (WMA) in comparison to mainland France. Using the same geographical referential in health studies will make possible to compare inequalities according to the health outcomes and to identify specific public health issues in each type of geographical context. The GeoClash classification can also be used as a proxy to assess the geographical context of the individuals in public health studies.

## **Supplementary information**

**Additional file 1.** Population density in the mainland France municipalities.

**Additional file 2.** Air pollution in the mainland France municipalities.

**Additional file 3.** Risk of pesticides exposure in the mainland France municipalities.

**Additional file 4.** Average income in the mainland France municipalities.

**Additional file 5.** Unemployment in the mainland France municipalities.

**Additional file 6.** Lower occupations in the mainland France municipalities.

**Additional file 7.** Unattached individuals in the mainland France municipalities.

**Additional file 8.** Level of education in the mainland France municipalities.

**Additional file 9.** Spatial accessibility to the hospitals in the mainland France municipalities.

**Additional file 10.** Spatial accessibility to the primary care practitioners in the mainland France municipalities.

## Data Availability

All data of the geographical scores and the GeoClasH classification presented in this study are available from the corresponding author on reasonable request.

## Abbreviations

AIP: Agricultural and Industrial Plains
APL: Accessibilité Potentielle Localisée (localized potential accessibility
GeoClasH: Geographical Classification for Health studies
IGN: Institut National Géographique (National Geographic Institute)
INERIS: Institut National de l'Environnement industriel et des RISques (National Institute for Industrial Environment and Risks)
INSEE: Institut National de la Statistique et des Etudes Economiques (National Institute of Statistics and Economic Studies)
IRIS: Îlots Regroupés pour l’Information Statistique (aggregated units for statistical information)
NO_2_: Nitrogen Dioxide
PCA: Principal Component Analysis
PM: Particulate Matters
PPD: Precarious Population Districts
RM: Rural Margins
RO: Residential Outskirts
SAE: Statistique Annuelle des Etablissements (Annual Statistics of the Facilities)
SMR: Standardized all-causes Mortality Rates
WMA: Wealthy Metropolitan Areas
