# Correction to: Beyond the map: evidencing the spatial dimension of health inequalities

**DOI:** 10.1186/s12942-020-00255-9

**Published:** 2021-01-04

**Authors:** Yohan Fayet, Delphine Praud, Béatrice Fervers, Isabelle Ray-Coquard, Jean-Yves Blay, Françoise Ducimetiere, Guy Fagherazzi, Elodie Faure

**Affiliations:** 1 Equipe EMS – Département de Sciences Humaines Et Sociales, Centre Léon Bérard, 28 rue Laennec, 69008 Lyon, France; 2 EA 7425 Health Services and Performance Research, Université de Lyon, Lyon, France; 3 Department Prevention Cancer Environment, Centre Léon Bérard, Lyon, France; 4 Inserm UA 08: Radiations, Défense, Santé, Environnement, Centre Léon Bérard, Lyon, France; 5 Department of Medical Oncology, Centre Léon Bérard, Université Claude Bernard, Lyon, France; 6 Digital Epidemiology and E-Health Research Hub, Department of Population Health, Luxembourg Institute of Health, Strassen, Luxembourg; 7 Center of Epidemiology and Population Health, UMR 1018, Inserm, Paris South, Paris Saclay University, Villejuif, France; 8 Gustave Roussy Institute, Villejuif, France

## Correction to: Int J Health Geogr (2020) 19:46 10.1186/s12942-020-00242-0

Unfortunately, the original version of the article [1] contained error in author names. The given and family names of all the authors were interchanged in original submission and in the final version published online. The author names are corrected with this correction.
