# Supplementary figures and images for: Beyond the map: evidencing the spatial dimension of health inequalities
Source: Int J Health Geogr. 2020 Nov 9;19:46. doi: 10.1186/s12942-020-00242-0 (PMC7727185; doi:10.1186/s12942-020-00242-0)

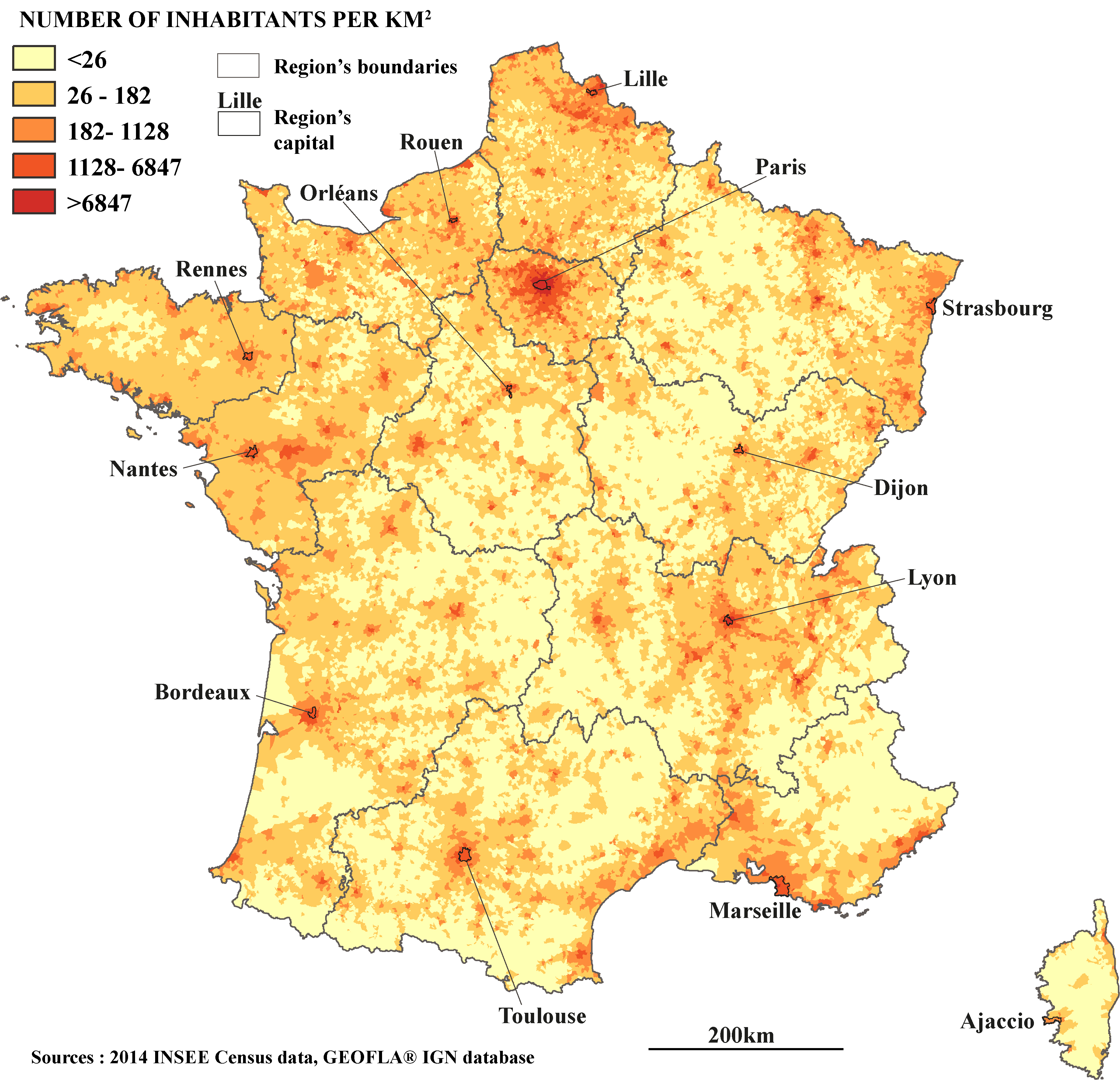

Supplement: Supplementary file 1 — Additional file 1. Population density in the mainland France municipalities. [file 12942_2020_242_MOESM1_ESM.jpg]

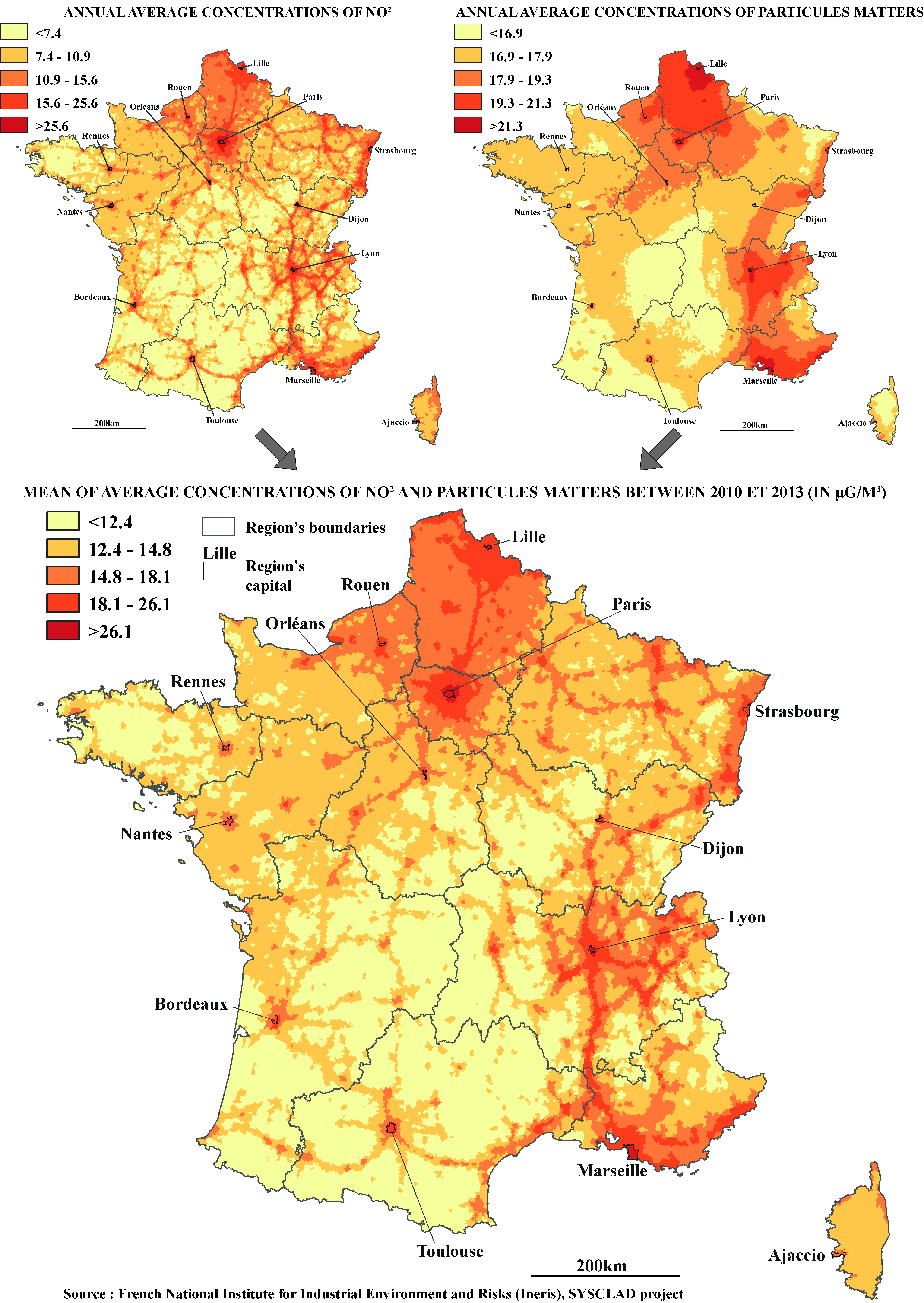

Supplement: Supplementary file 2 — Additional file 2. Air pollution in the mainland France municipalities. [file 12942_2020_242_MOESM2_ESM.jpg]

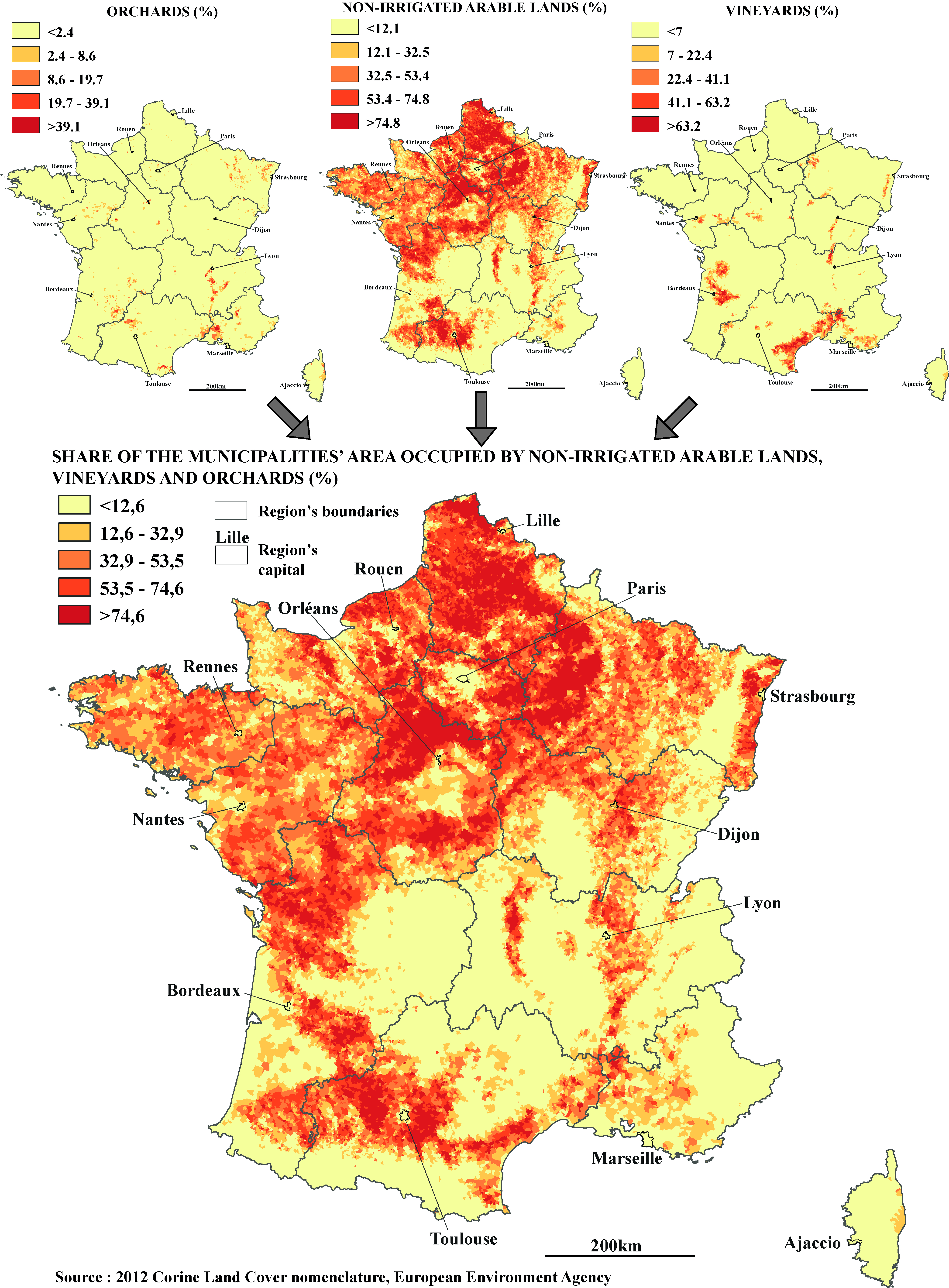

Supplement: Supplementary file 3 — Additional file 3. Risk of pesticides exposure in the mainland France municipalities. [file 12942_2020_242_MOESM3_ESM.jpg]

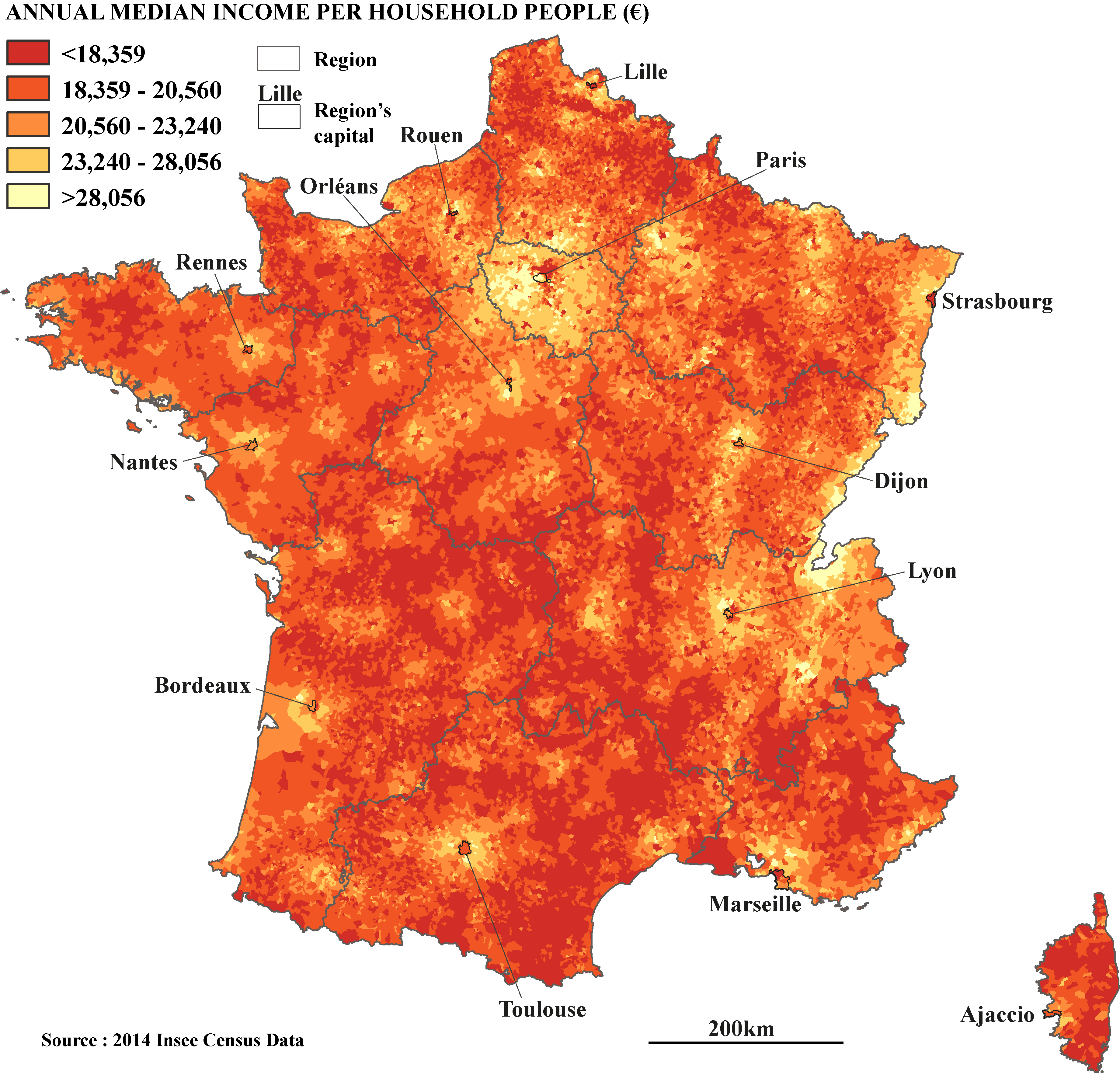

Supplement: Supplementary file 4 — Additional file 4. Average income in the mainland France municipalities. [file 12942_2020_242_MOESM4_ESM.jpg]

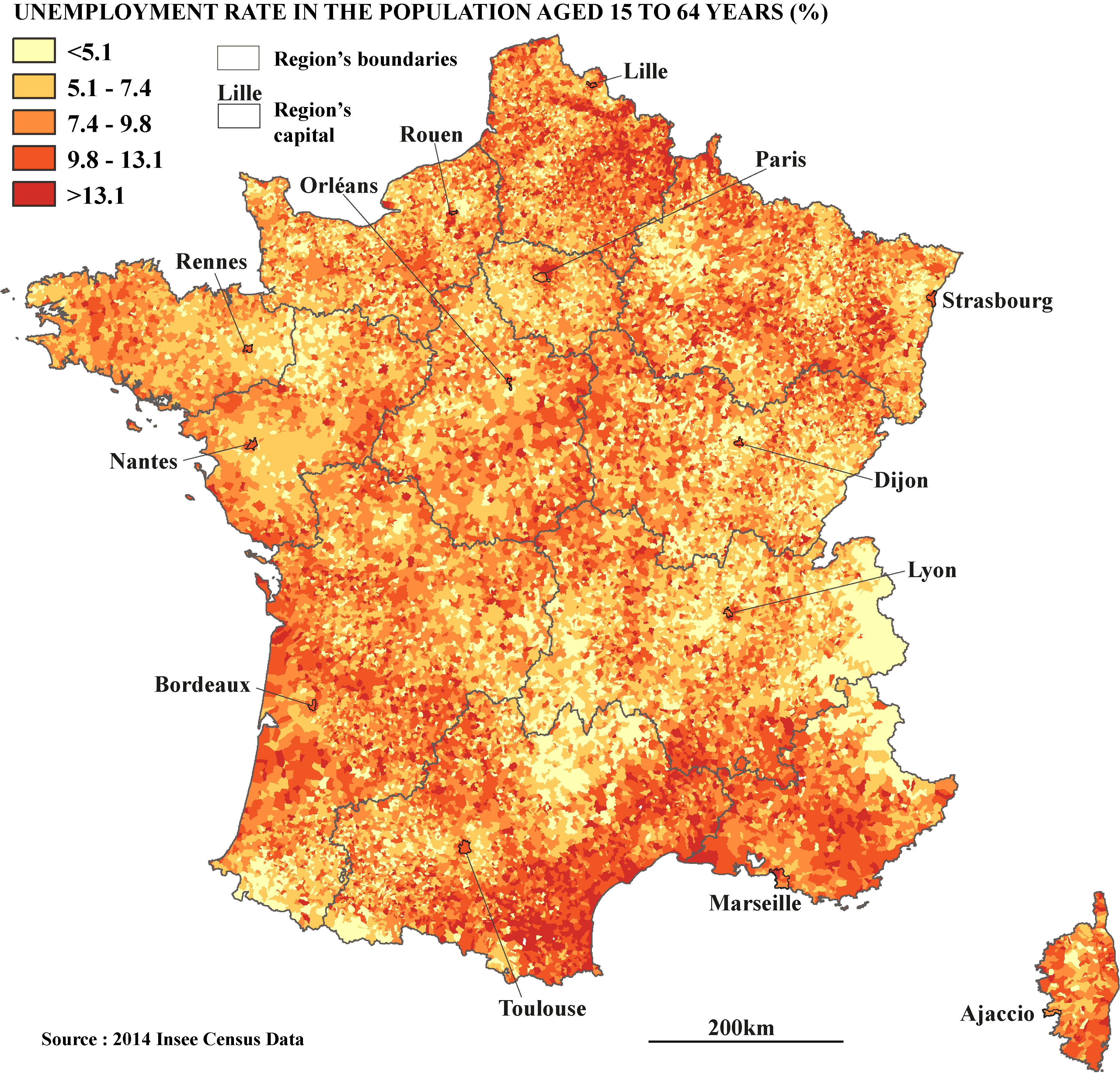

Supplement: Supplementary file 5 — Additional file 5. Unemployment in the mainland France municipalities. [file 12942_2020_242_MOESM5_ESM.jpg]

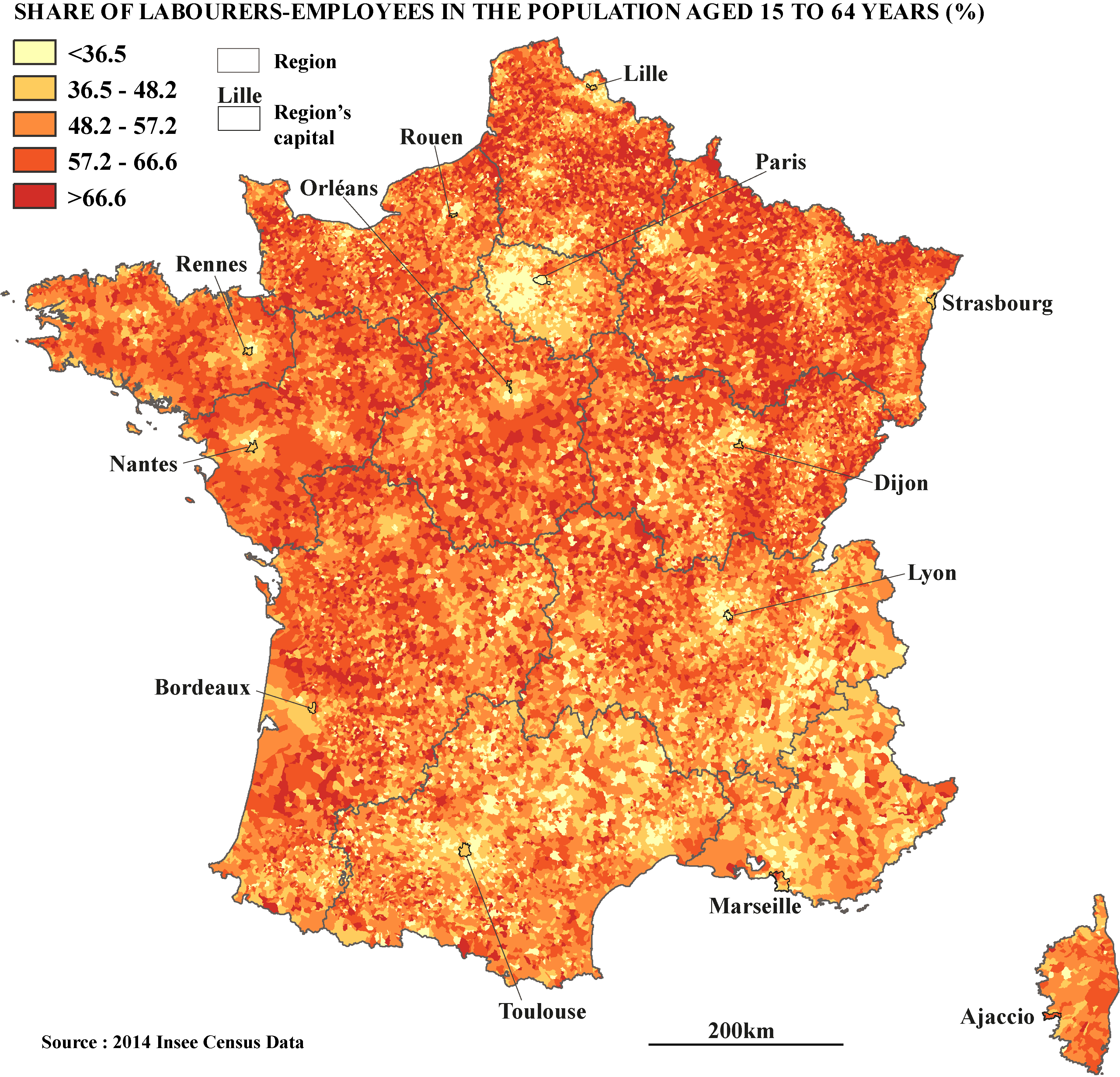

Supplement: Supplementary file 6 — Additional file 6. Lower occupations in the mainland France municipalities. [file 12942_2020_242_MOESM6_ESM.jpg]

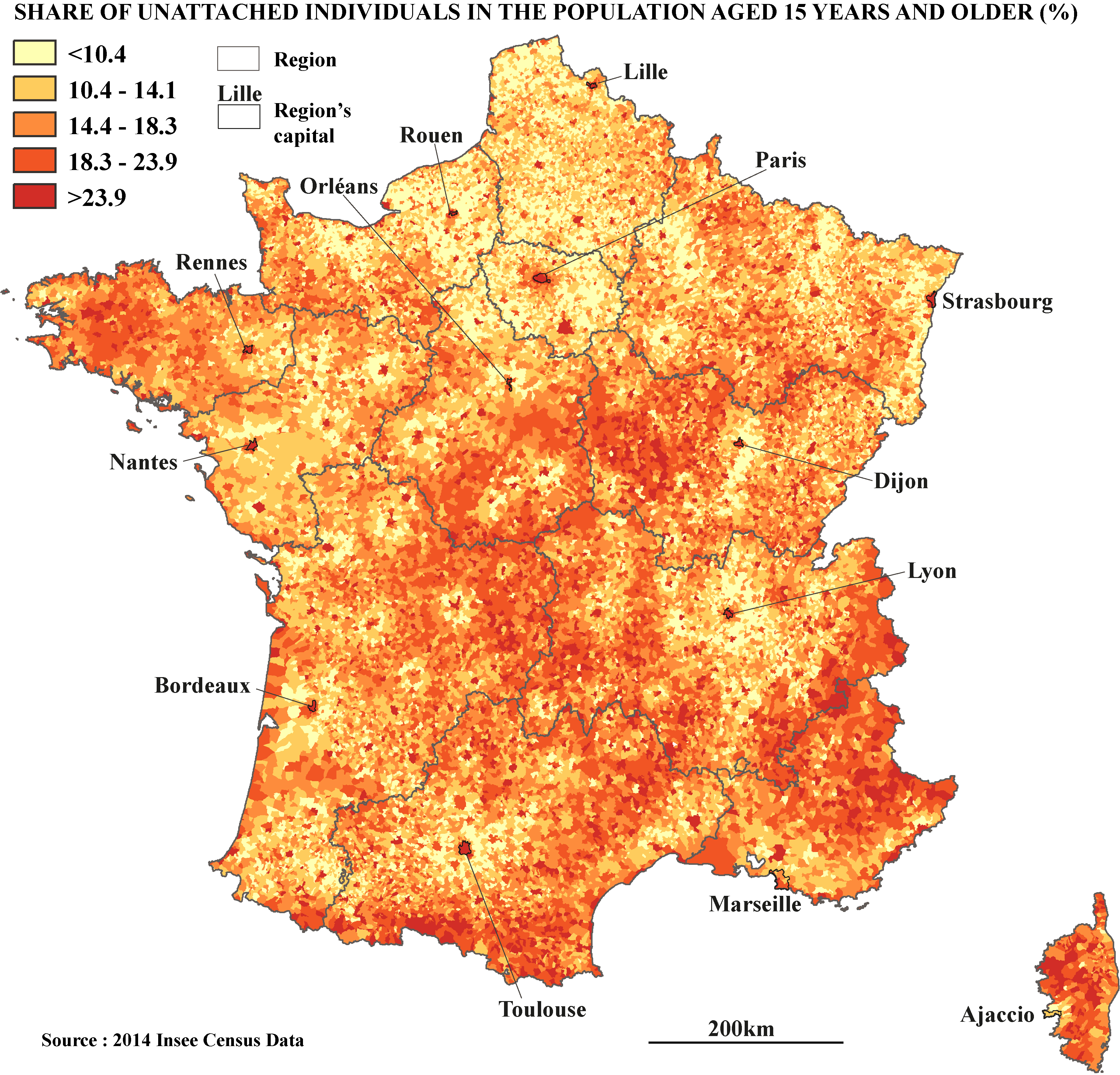

Supplement: Supplementary file 7 — Additional file 7. Unattached individuals in the mainland France municipalities. [file 12942_2020_242_MOESM7_ESM.jpg]

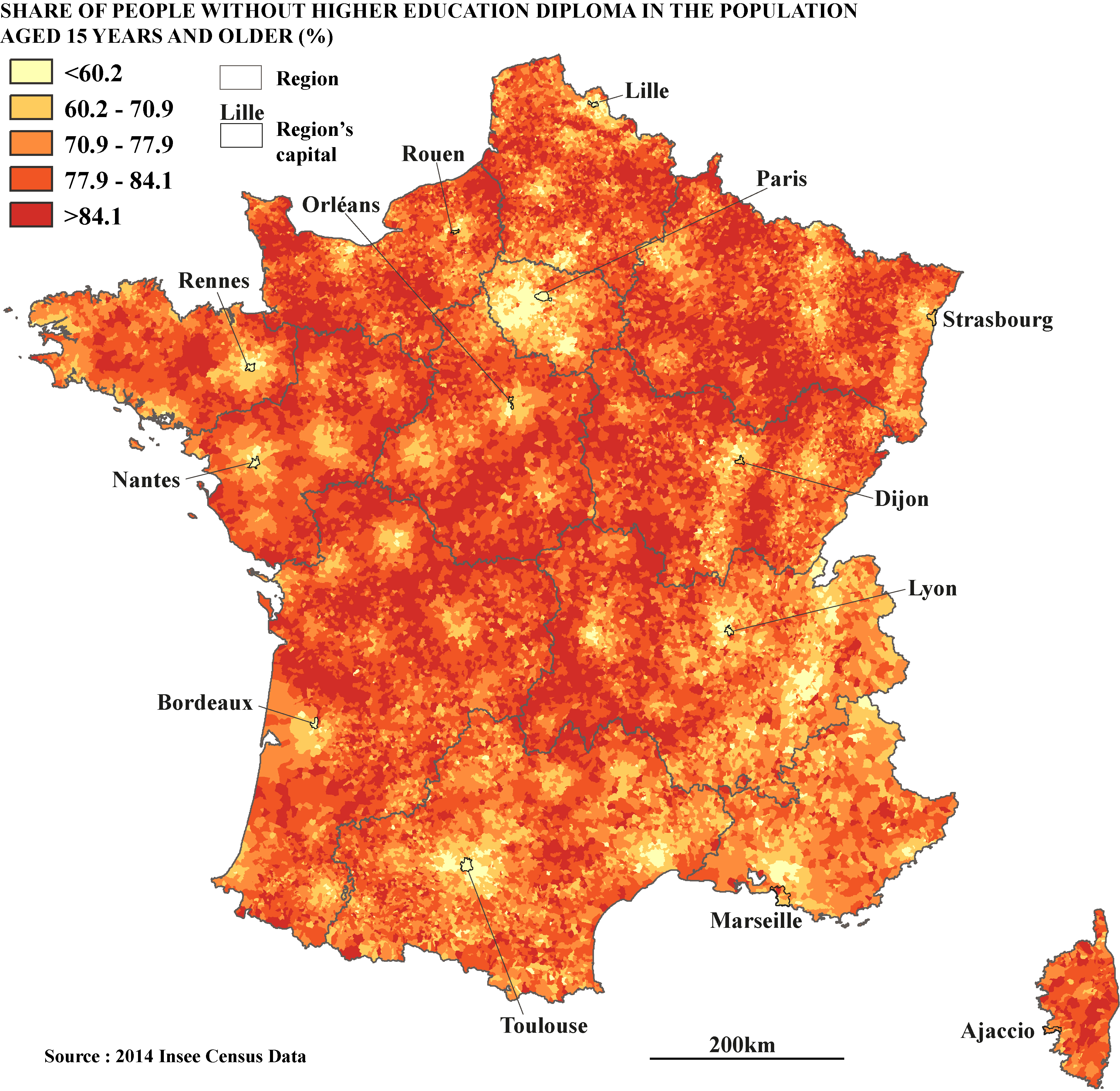

Supplement: Supplementary file 8 — Additional file 8. Level of education in the mainland France municipalities. [file 12942_2020_242_MOESM8_ESM.jpg]

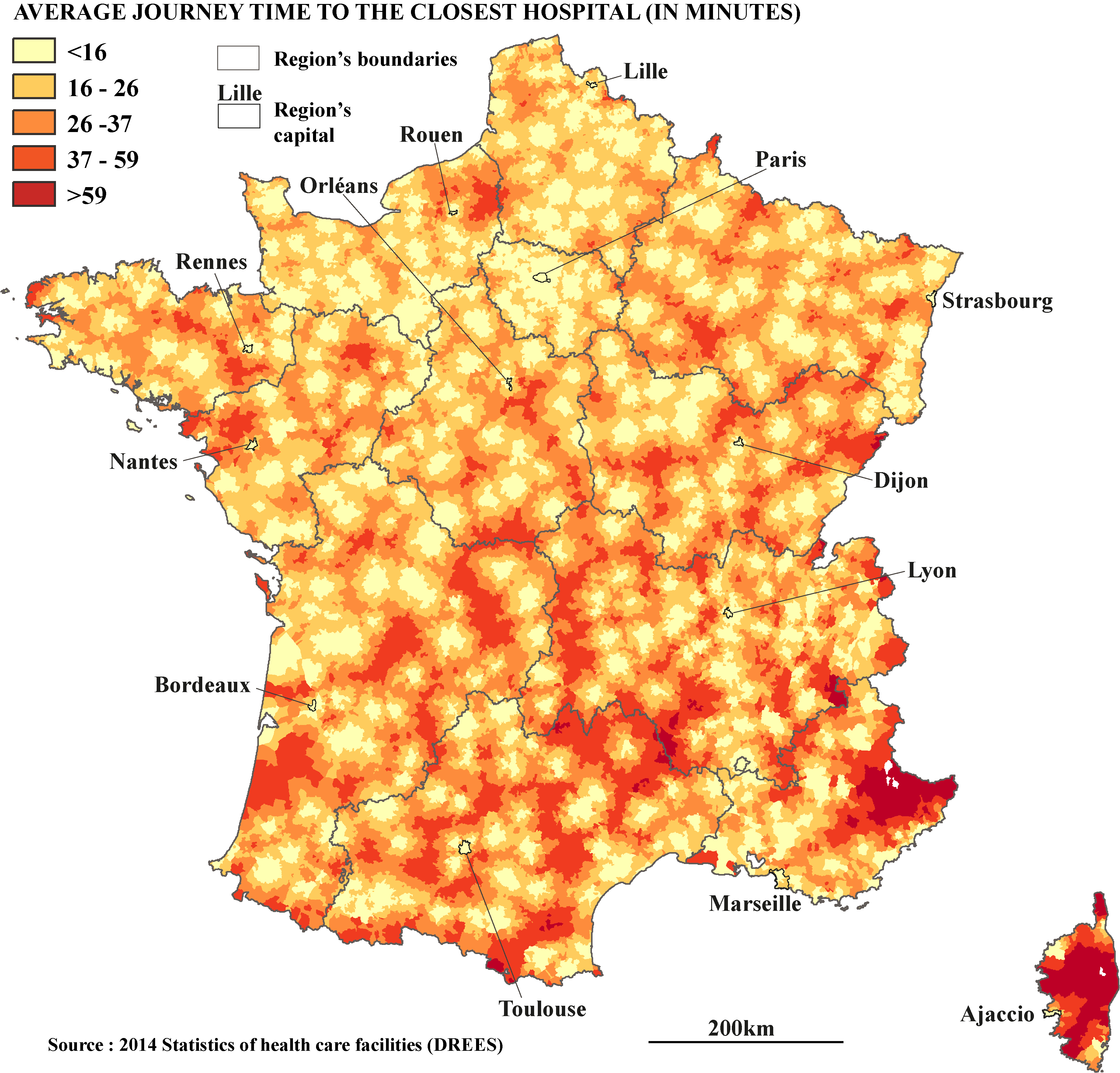

Supplement: Supplementary file 9 — Additional file 9. Spatial accessibility to the hospitals in the mainland France municipalities. [file 12942_2020_242_MOESM9_ESM.jpg]

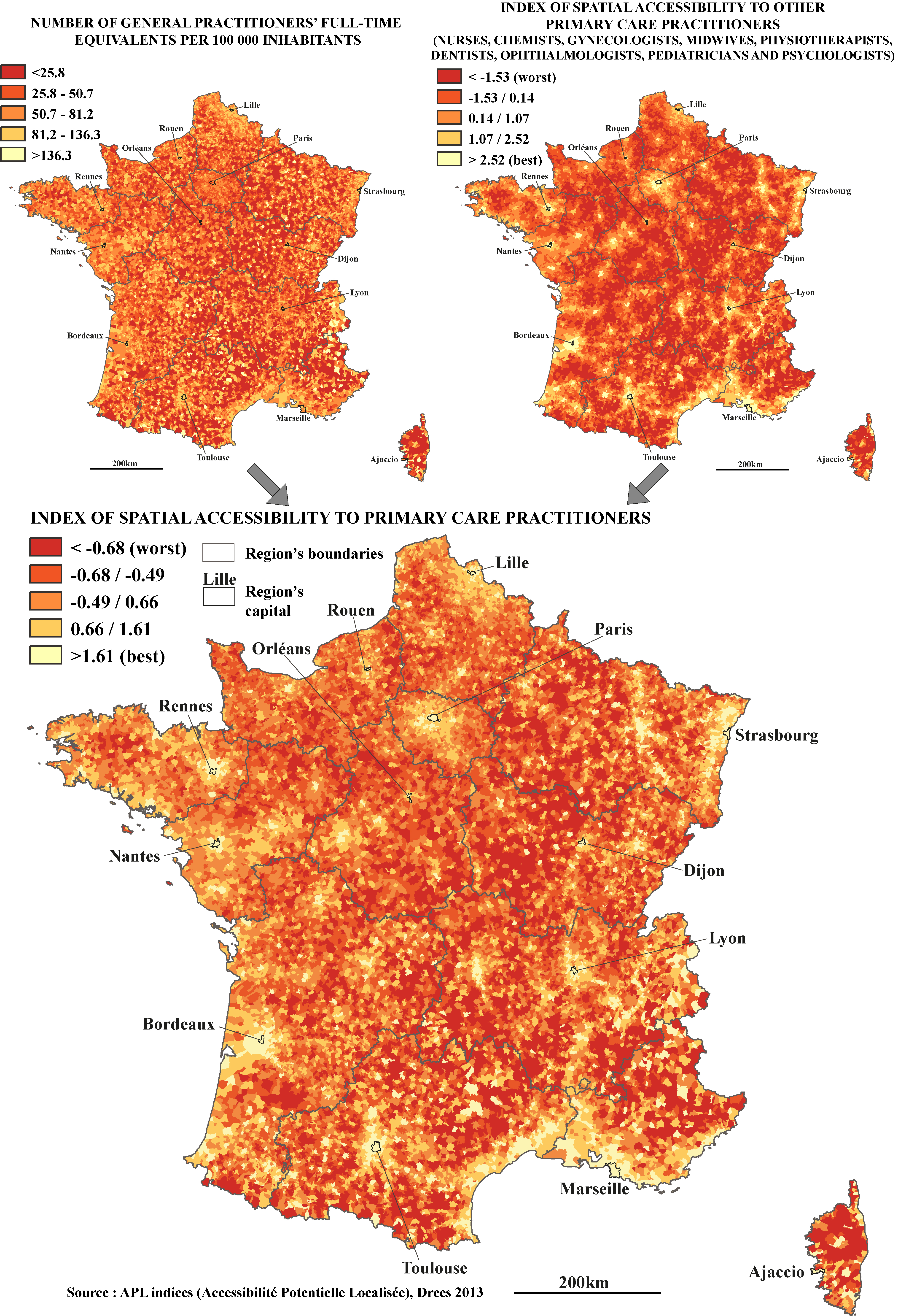

Supplement: Supplementary file 10 — Additional file 10. Spatial accessibility to the primary care practitioners in the mainland France municipalities. [file 12942_2020_242_MOESM10_ESM.jpg]
